# Transcriptomic Analysis Revealed Antimicrobial Mechanisms of *Lactobacillus rhamnosus* SCB0119 against *Escherichia coli* and *Staphylococcus aureus*

**DOI:** 10.3390/ijms232315159

**Published:** 2022-12-02

**Authors:** Huan Peng, Gang Zhou, Xi-Miao Yang, Guo-Jun Chen, Hai-Bin Chen, Zhen-Lin Liao, Qing-Ping Zhong, Li Wang, Xiang Fang, Jie Wang

**Affiliations:** 1 College of Food Science, South China Agricultural University, Guangzhou 510642, China; 2 Guangdong Open Laboratory of Applied Microbiology, Guangdong Provincial Key Laboratory of Microbial Culture Collection and Application, State Key Laboratory of Applied Microbiology Southern China, Guangdong Institute of Microbiology, Guangdong Academy of Sciences, Guangzhou 510070, China

**Keywords:** *Lactobacillus rhamnosus*, cell-free culture supernatant, *Escherichia coli*, *Staphylococcus aureus*, antimicrobial activity, transcriptomic analysis

## Abstract

Lactic acid bacteria were reported as a promising alternative to antibiotics against pathogens. Among them, *Lactobacillus rhamnosus* could be used as probiotics and inhibit several pathogens, but its antibacterial mechanisms are still less known. Here, *L. rhamnosus* SCB0119 isolated from fermented pickles could inhibit bacterial growth or even cause cell death in *Escherichia coli* ATCC25922 and *Staphylococcus aureus* ATCC6538, which was mainly attributed to the cell-free culture supernatant (CFS). Moreover, CFS induced the accumulation of reactive oxygen species and destroyed the structure of the cell wall and membrane, including the deformation in cell shape and cell wall, the impairment of the integrity of the cell wall and inner membrane, and the increases in outer membrane permeability, the membrane potential, and pH gradient in *E. coli* and *S. aureus*. Furthermore, the transcriptomic analysis demonstrated that CFS altered the transcripts of several genes involved in fatty acid degradation, ion transport, and the biosynthesis of amino acids in *E. coli*, and fatty acid degradation, protein synthesis, DNA replication, and ATP hydrolysis in *S. aureus*, which are important for bacterial survival and growth. In conclusion, *L. rhamnosus* SCB0119 and its CFS could be used as a biocontrol agent against *E. coli* and *S. aureus*.

## 1. Introduction

Foodborne pathogens, including *Escherichia coli* and *Staphylococcus aureus*, cause foodborne diseases and bring serious harm to human health [1,2,3]. Approximately 2.8 billion people worldwide suffer from diarrhea due to foodborne illness, and about 3000 people die each year from illness by ingesting food contaminated with foodborne pathogens in the United States [4]. Existing widely in food, water, food-producing animals, and so on [5], *E. coli* can cause various human diseases ranging from gastroenteritis, such as diarrhea, to extra-intestinal infections, including those of the urinary tract, bloodstream, and central nervous system [6,7]. For example, Shiga toxin-producing *E. coli* caused 2,801,000 acute illnesses annually, 3890 cases of hemolytic uremic syndrome, 270 cases of permanent end-stage renal disease, and 230 deaths, according to databases from 21 countries published between 1 January 1990 and 30 April 2012 [8]. As one of the main foodborne pathogens contaminating ready-to-eat products, meat, egg, dairy products, cream-filled pastries, and cakes [9] and existing in food production animals [10], *S. aureus* could produce exotoxins and cause food poisoning with the symptoms including nausea, vomiting, and abdominal cramps with or without diarrhea [11]. Additionally, *S. aureus* is a leading cause of bacteremia, which harbors high rates in the first year of life, a low incidence through young adulthood, and a gradual rise in incidence with advancing age, and the population incidence of *S. aureus* bacteremia is 10–30 per 100,000 person-years in industrialized countries [12]. To prevent the contamination of foodborne pathogens, antibiotics and/or chemical preservatives are widely used in animal husbandry and food manufacturing, but the excessive and unreasonable use of antibiotics and chemical preservatives resulted in new hazards to human health, such as the accumulation of chemical residues in food and feed chain [13], the development of antibiotic resistance in bacteria, and environmental pollution [14]. Thus, it is necessary to find an effective and alternative way to replace antibiotics and chemical preservatives. 

Lactic acid bacteria are regarded as a promising alternative to antibiotics against the contamination of pathogens due to high-effective antibacterial activities, various antimicrobial substances, beneficial effects on human and animal health, and so on [15,16]. Among them, *Lactobacillus rhamnosus* is generally considered to be beneficial to human health and commonly used as a probiotic [17]. Previous studies showed that *L. rhamnosus* could inhibit the growth of several pathogens, such as *S. aureus*, *E. coli*, *Candida albicans*, *Shigella sonnei*, *Salmonella enteritidis*, *Pseudomonas fluorescens*, *Pseudomonas putida*, and *Listeria monocytogene*, and its antibacterial activities differed among different *L. rhamnosus* strains [18,19]. However, it was still little known about the antibacterial mechanisms of *L. rhamnosus*, though it was reported to inhibit the growth of *S. enteritidis* by elevating the reactive oxygen species level and destructing bacterial membrane [20] and inhibiting the growth of *S. aureus* by increasing cell permeability [21]. In this study, we evaluated the potential antibacterial effects of *L. rhamnosus* SCB0119 isolated from fermented pickles against *E. coli* and *S. aureus* by investigating the changes in bacterial growth and cell structures. Moreover, transcriptomic analysis was used to unveil the underlying mechanism of actions. The findings will be helpful in preventing the contamination of *E. coli* and *S. aureus* and developing a novel preventative strategy against pathogens not limited to *E. coli* and *S. aureus*.

## 2. Results

### 2.1. Reduction in Bacterial Growth of E. coli and S. aureus Caused by L. rhamnosus SCB0119

**Figure 1:**
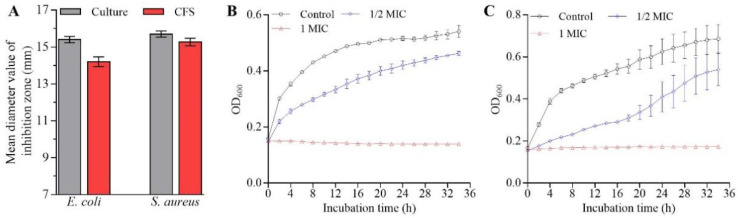
Antimicrobial activity of *L. rhamnosus* SCB0119 against *E. coli* ATCC25922 and *S. aureus* ATCC6538. (**A**) The mean diameters of inhibition zones after *E. coli* ATCC25922 and *S. aureus* ATCC6538 were incubated with *L. rhamnosus* SCB0119 culture solution and CFS for 12 h, respectively. (**B**,**C**) Effects of CFS on the growth of *E. coli* ATCC25922 and *S. aureus* ATCC6538, respectively. Error bars: SD of the mean from three repeated assays.

The *L. rhamnosus* SCB0119 culture solution and cell-free culture supernatant (CFS) showed antimicrobial activity against *E. coli* and *S. aureus* with mean inhibition zone diameters of 15.4 and 14.2 mm for *E. coli* ATCC25922, and 15.7 and 15.27 mm for *S. aureus* ATCC6538, respectively (Figure 1A). However, resuspended bacterial cells displayed no antagonistic activity (no inhibition zone) against *E. coli* ATCC25922 and *S. aureus* ATCC6538. These results demonstrated the antimicrobial activity of *L. rhamnosus* SCB0119 was mainly attributed to its metabolites.

To further investigate the effects of *L. rhamnosus* SCB0119 on bacterial growth and its potential mechanisms, CFS was chosen to conduct the following studies. The minimum inhibitory concentrations (MICs) of CFS against *E. coli* ATCC25922 and *S. aureus* ATCC6538 were 3.91 and 7.81 mg/mL, respectively. Obviously, 1 MIC of CFS completely inhibited the growth of *E. coli* ATCC25922 (Figure 1B) and *S. aureus* ATCC6538 (Figure 1C). Moreover, the reduction in bacterial growth of *E. coli* ATCC25922 and *S. aureus* ATCC6538 started at the early stationary phase, and the time needed to reach a plateau was delayed by 6 h for *E. coli* ATCC25922 (Figure 1B) and 4 h for *S. aureus* ATCC6538 (Figure 1C), when 1/2 MIC of CFS was added into Luria Bertani (LB) medium. 

### 2.2. Effects of CFS on the Production of Intracellular ATP and Reactive Oxygen Species in E. coli and S. aureus

**Figure 2:**
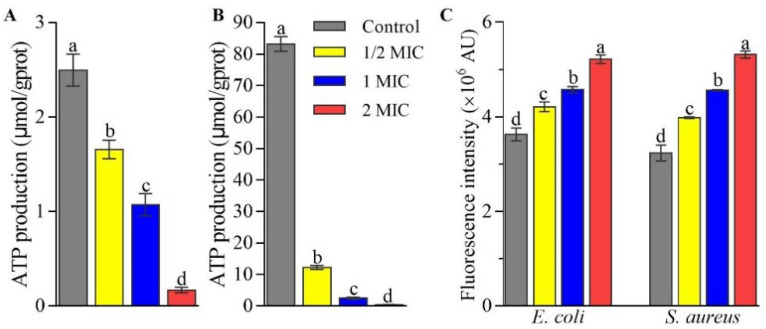
Effects of CFS from *L. rhamnosus* SCB0119 on the production of ATP (**A**,**B**) and ROS (**C**) in *E. coli* ATCC25922 (**A**,**C**) and *S. aureus* ATCC6538 (**B**,**C**). ROS production was detected using DCFH-DA staining. Different lowercase letters indicate significant differences among the treatments (*p* < 0.05). Error bars: SD of the mean from three repeated assays.

CFS dose-dependently inhibited the production of intracellular ATP in *E. coli* ATCC25922 and *S. aureus* ATCC6538. In comparison to 2.49 and 83.22 nmol/mg protein on average for *E. coli* ATCC25922 (Figure 2A) and *S. aureus* ATCC6538 (Figure 2B), respectively, in the control group, the ATP production decreased by 33.68% and 85.27% in 1/2 MIC group, 57.01% and 96.95% in 1 MIC group, and 93.31% and 99.49% in 2 MIC group, respectively, suggesting CFS inhibited *E. coli* ATCC25922 and *S. aureus* ATCC6538 growth or even caused death because the decreases in intracellular ATP levels mean that cells undergo apoptosis, necrosis, or are in a toxic state [22].

Moreover, CFS induced oxidative stress in *E. coli* ATCC25922 and *S. aureus* ATCC6538 cells. As shown in Figure 2C, the fluorescence intensities using DCFH-DA staining in *E. coli* ATCC25922 and *S. aureus* ATCC6538 cells increased by 16.10% and 23.09% in 1/2 MIC group, 26.12% and 41.03% in 1 MIC group, and 43.90% and 64.36% in 2 MIC group, respectively, compared with those in the control group, suggesting CFS increased the reactive oxygen species (ROS) levels in a dose-dependent manner. 

### 2.3. Changes in Cell Morphology and Structure of E. coli and S. aureus Caused by CFS

**Figure 3:**
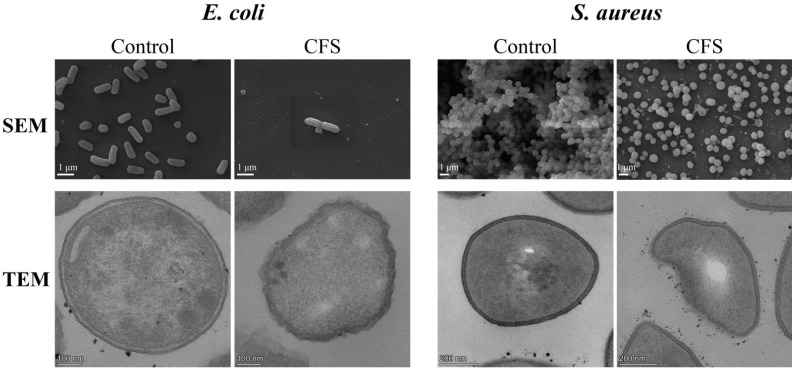
SEM and TEM images after *E. coli* ATCC25922 and *S. aureus* ATCC6538 cells were treated with 1/2 MIC of CFS from *L. rhamnosus* SCB0119.

Cell morphology and structure of *E. coli* ATCC25922 and *S. aureus* ATCC6538 were affected by CFS. Firstly, scanning electron microscopy (SEM) images showed that fewer cells and more shrunk cells were observed in the CFS group than that in the control group (Figure 3). Moreover, *S. aureus* ATCC6538 cells became dispersed rather than clustered after being treated with CFS (Figure 3). Additionally, transmission electron microscopy (TEM) images displayed that *E. coli* ATCC25922 and *S. aureus* ATCC6538 cells treated with CFS were deformed, ruptured, and less distinctly outlined compared with untreated cells (Figure 3). 

**Figure 4:**
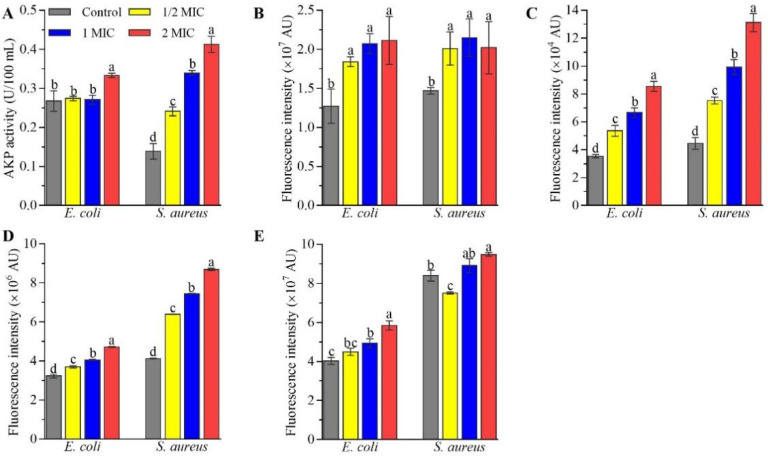
Effects of CFS from *L. rhamnosus* SCB0119 on AKP contents (**A**), outer membrane permeability (**B**), inner membrane integrity (**C**), membrane potential (**D**) and pH gradients (**E**) in *E. coli* ATCC25922 and *S. aureus* ATCC6538. Changes in the outer membrane permeability, inner membrane integrity, membrane potential, and pH gradients were assayed using NPN, PI, DiBAC4(3), and BCECF-AM staining, respectively. Different lowercase letters indicate significant differences among the treatments (*p* < 0.05). Error bars: SD of the mean from three repeated assays.

Secondly, CFS impaired cell wall integrity based on alkaline phosphatase (AKP) activity. As shown in Figure 4A, the AKP contents in *S. aureus* ATCC6538 cells increased by 73.81% in the 1/2 MIC group, 144.76% in the 1 MIC group, and 197.62% in the 2 MIC group, and those in *E. coli* ATCC25922 cells increased 24.69% in 2 MIC group, compared with those in the control group, though CFS at 1/2 and 1 MIC did not change the AKP contents in *E. coli* ATCC25922 cells. 

Moreover, CFS increased outer membrane permeability and impaired inner membrane integrity. The fluorescence intensities using 1-N-phenylnaphthylamine (NPN) staining, which has become a common fluorescent detection signal to evaluate the extent of bacterial outer membrane damage, increased by 36.92–66.18% in *E. coli* ATCC25922 and *S. aureus* ATCC6538 cells treated with CFS at different concentrations, compared with those in untreated cells (Figure 4B). Additionally, the fluorescence intensities using propidium iodide (PI) staining in *E. coli* ATCC25922 and *S. aureus* ATCC6538 cells increased by 51.39% and 69.13% in the 1/2 MIC group, 88.44% and 123.12% in the 1 MIC group, and 141.69% and 194.72% in the 2 MIC group (Figure 4C), respectively, compared with those in the control group, suggesting CFS impaired inner membrane integrity in a dose-dependent manner.

Accompanied by the destruction of cell membrane integrity, CFS increased the membrane potential (ΔΨ) and pH gradient (ΔpH). As shown in Figure 4D, the fluorescence intensities using DiBAC4(3) staining in *E. coli* ATCC25922 and *S. aureus* ATCC6538 cells increased by 14.55% and 55.03% in the 1/2 MIC group, 25.83% and 80.51% in the 1 MIC group, and 46.30% and 110.69% in the 2 MIC group, respectively, compared with those in the control group, suggesting CFS caused depolarization of *E. coli* ATCC25922 and *S. aureus* ATCC6538 cells. Similarly, the fluorescence intensities using BCECF-AM staining in *E. coli* ATCC25922 cells increased by 11.73% in the 1/2 MIC group, 22.73% in the 1 MIC group, and 45.31% in the 2 MIC group, and those in *S. aureus* ATCC6538 cells increased by 6.12% and 12.92 in the 1 MIC and 2 MIC groups, respectively, but decreased by 10.57% in the 1/2 MIC group (Figure 4E), compared with those in the control group. 

### 2.4. Transcriptomic Analysis of E. coli and S. aureus Cells in Response to CFS

**Figure 5:**
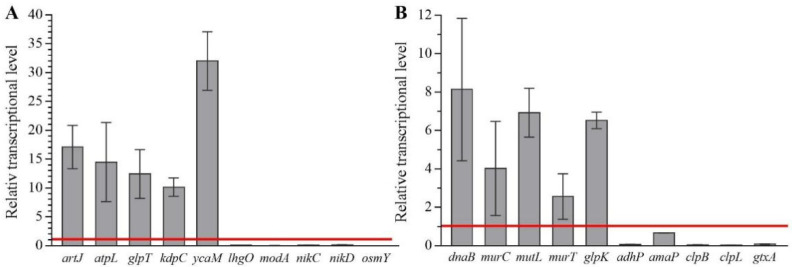
Fold change of selected genes by qRT-PCR after bacterial cells were treated with CFS from *L. rhamnosus* SCB0119 in *E. coli* ATCC25922 (**A**) and *S. aureus* ATCC6538 (**B**). The bacterial 16S rRNA was used as an internal standard. The red line at value 1 represented the expression of chosen genes in the control groups. Error bars: SD of the mean from three repeated assays.

In *E. coli* ATCC25922, the RNA sequencing results showed that the raw sequence outputs were 32823244, 35152464, 35876796, and 33148174 reads with 88.70%, 90.15%, 88.54%, and 88.21% total matches for the two CFS-free experiments and two CFS experiments, respectively (Table S2). Compared with those in CFS-free experiments, 1/2 MIC of CFS differentially upregulated the expression of 327 genes and downregulated the expression of 70 genes (Table S3). For *S. aureus* ATCC6538, the raw sequence outputs were 25524088, 21597232, 26838638, and 24129642 reads with 91.41%, 86.25%, 90.26%, and 80.69% total matches for the two CFS-free experiments and two CFS experiments, respectively (Table S2). When treated with 1/2 MIC of CFS, 814 genes were significantly up-regulated, and 233 genes were differentially down-regulated (Table S4). Moreover, all five upregulated and five downregulated genes selected for evaluation in *E. coli* ATCC25922 and *S. aureus* ATCC6538 were differentially expressed in response to CFS (Figure 5), confirming the reliability of the transcriptome data.

**Figure 6:**
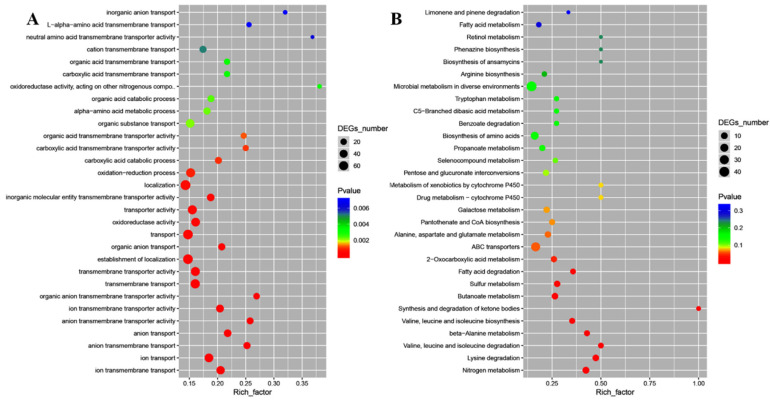
The GO enrichment scatter plot (**A**) and KEGG enrichment scatter plot (**B**) of differentially expressed genes treated with CFS from *L. rhamnosus* SCB0119 in *E. coli* ATCC25922.

**Figure 7:**
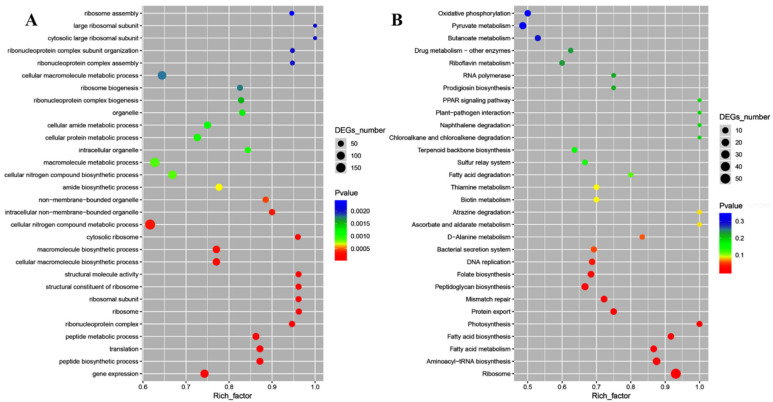
The GO enrichment scatter plot (**A**) and KEGG enrichment scatter plot (**B**) of differentially expressed genes treated with CFS from *L. rhamnosus* SCB0119 in *S. aureus* ATCC6538.

Moreover, gene ontology (GO) enrichment analysis classified the functions of the differentially expressed genes (DEGs) into biological process, cellular component, and molecular function, which included 57, 1, and 23 for *E. coli* ATCC25922, and 44, 15, and 4 for *S. aureus* ATCC6538, respectively. In *E. coli* ATCC25922, ion transmembrane transport and ion transport, outer membrane-bounded periplasmic space, and anion transmembrane transporter activity and ion transmembrane transporter activity were dominant for biological process, cellular component, and molecular function, respectively (Figure 6A). Kyoto encyclopedia of genes and genomes (KEGG) enrichment analysis demonstrated that DEGs were mapped into nitrogen metabolism, amino acid metabolism, synthesis and degradation of ketone bodies, sulfur metabolism, ABC transporters, carbohydrate metabolism, metabolism of cofactors and vitamins, benzoate degradation, microbial metabolism in diverse environments, drug metabolism-cytochrome P450, and fatty acid metabolism (Figure 6B), which were crucial for lipid metabolism, amino acid metabolism, cellular structure and processes, and membrane transport. For *S. aureus* ATCC6538, GO enrichment analysis showed that gene expression and peptide biosynthetic process, ribonucleoprotein complex and ribosome, and structural molecule activity and structural constituent of ribosome were dominant for biological process, cellular component, and molecular function, respectively (Figure 7A). KEGG enrichment analysis demonstrated that DEGs were mapped into the ribosome, aminoacyl-tRNA biosynthesis, peptidoglycan biosynthesis, protein export, mismatch repair, DNA replication, fatty acid metabolism, bacterial secretion system, metabolism of cofactors and vitamins, the PPAR signaling pathway, RNA polymerase, carbohydrate metabolism, and oxidative phosphorylation (Figure 7B), which are crucial for lipid metabolism, energy metabolism, translation, and replication and repair. These data implied that the targeted genes of CFS in *E. coli* might differ from those in *S. aureus*.

To illuminate the antimicrobial mechanisms of CFS against *E. coli* and *S. aureus*, we further analyzed the DEGs involved in the biosynthesis of amino acids, ABC transporters, and fatty acid degradation in *E. coli* ATCC25922, and ribosome, aminoacyl-tRNA biosynthesis, DNA replication, oxidative phosphorylation, and fatty acid degradation in *S. aureus* ATCC6538. In *E. coli* ATCC25922, CFS increased the expressions of *ilvN*, *ilvI*, *leuA*, *leuB*, and *lysC* involved in the biosynthesis of amino acids by 2.08–8.75-fold and decreased the expressions of ABC transporter *nikE*, *nikD*, *nikC*, *nikB*, *nikA*, and *fepD* by 52.89–81.45% and the expression of *adhP* involved in fatty acid degradation by 27.94% (Table 1). Moreover, the expression of *osmY* encoding one periplasmic protein was down-regulated by 18.14% (Table S3). In *S. aureus* ATCC6538, CFS upregulated the expression of genes encoding 30S small subunits, 50S large subunits, and aminoacyl-tRNA by 3.66–104.47 folds and the expressions of *atpD*, *atpA*, *atpE*, and *atpB* related to oxidative phosphorylation by 5.48–16.79 folds, but decreased the expressions of *adhP* and EKM74_RS04795 related to DNA replication by 38.05% and 53.22%, respectively (Table 2). Moreover, the expressions of *frr* and *rbfA* related to ribosome cofactor were up-regulated by 3.19- and 12.50-fold (Table S4), respectively.

## 3. Discussion

Lactic acid bacteria were reported as a promising alternative to antibiotics against several pathogens [18]. Here, *L. rhamnosus* SCB0119 could inhibit the growth of *E. coli* ATCC25922 and *S. aureus* ATCC6538. Moreover, its CFS, the main component for antibacterial activity, inhibited bacterial growth or even caused death, induced oxidative stress, and impaired cell shape and structure, as discussed below.

CFS from *L. rhamnosus* SCB0119 reduced bacterial growth of *E. coli* ATCC25922 and *S. aureus* ATCC6538. According to the diameter of the inhibition zone, CFS from *L. rhamnosus* SCB0119 exhibited greater antibacterial activity against *E. coli* ATCC25922 and *S. aureus* ATCC6538 than that from *L. rhamnosus* XN2 (7.2 ± 0.5 mm for *E. coli* CICC10302 and 15.1 ± 1.2 mm for *S. aureus* CICC10384) and LMG 23,522 (8.9 ± 0.2 mm for *E. coli* O157:H7 and 11.3 ± 0.4 mm for *S. aureus*) [21,23]. Additionally, the decreases in intracellular ATP levels suggested that CFS from *L. rhamnosus* SCB0119 might cause the death of *E. coli* and *S. aureus* cells [22]. The cell death of pathogens caused by *L. rhamnosus* was also reported in *S. typhimurium* treated with *L. rhamnosus* SQ511 [20].

Moreover, ROS production plays an important role in commencing cessation of the cell during the initiation of cell death [24]. Here, CFS from *L. rhamnosus* SCB0119 accumulated the ROS levels in *E. coli* ATCC25922 and *S. aureus* ATCC6538, which was similar to the increases of ROS production in *S. typhimurium* caused by CFS from *L. rhamnosus* SQ511 [20]. The high level of ROS production causes oxidative stress, which is closely associated with adverse effects on cellular components and causes cell death [25,26].

Furthermore, CFS from *L. rhamnosus* SCB0119 destroyed the cell wall, outer membrane, and plasma membrane. First, the cell wall is the first line to protect cells, including the cell membrane and organelles [27,28]. When the cell wall is damaged, AKP existing between the cell wall and the membrane of bacteria leaks into the extracellular milieu, causing an increase in extracellular AKP activity [29]. In this study, CFS increased AKP contents in *E. coli* ATCC25922 and *S. aureus* ATCC6538, suggesting the cell wall of *E. coli* and *S. aureus* was impaired by CFS from *L. rhamnosus* SCB0119. The impairment of the cell wall might affect the cell membrane. Generally, effective antibacterial compounds must penetrate or destroy the plasma membrane of the pathogen [30]. The increases in fluorescence intensity of PI staining, ΔΨ, and ΔpH indicated that CFS from *L. rhamnosus* SCB0119 destroyed the integrity of the cell membrane and improved the cell membrane permeability in *E. coli* ATCC25922 and *S. aureus* ATCC6538, which resulted in the dissipation of membrane ΔΨ and ΔpH and depolarization of the cytoplasmic membrane [31,32]. Changes in PI staining, ΔΨ, and ΔpH were consistent with that in *E. coli* cells treated with a bacteriocin produced by *L. rhamnosus* 1.0320 [32]. Additionally, changes in PI staining coincided with that in *S. aureus* cells conditioned with CFS from *L. rhamnosus* XN2 [21], and the depolarization of cell membrane was in agreement with that in *S. enteritidis* caused by CFS from *L. rhamnosus* SQ511 [20]. Besides, disruption of membrane integrity leads to intracellular ATP leakage, which is also responsible for the decreased intracellular ATP concentration [33]. The elimination of the transmembrane electrochemical gradient might also lead to the loss of the ability of bacterial cells to synthesize ATP [34]. Moreover, the outer membrane is important for barrier function and nutrient flow [35]. The increases in the amount of NPN staining demonstrated that CFS from *L. rhamnosus* SCB0119 increased the permeability of the outer membrane in *E. coli* ATCC25922 and *S. aureus* ATCC6538, which concurred with the findings found in *E. coli* cells treated with a bacteriocin produced by *L. rhamnosus* 1.0320 [32]. These data indicated that CFS from *L. rhamnosus* SCB0119 destroyed the integrity of the cell wall and membrane, increased the permeability of the cell membrane, released the cell contents, and induced cell death in *E. coli* and *S. aureus*.

Additionally, CFS from *L. rhamnosus* SCB0119 affected the transcriptional expression of genes involved in fatty acid degradation, biosynthesis of amino acids, ABC transporters, ribosome, aminoacyl-tRNA biosynthesis, DNA replication, and/or oxidative phosphorylation in *E. coli* ATCC25922 and *S. aureus* ATCC6538. Firstly, *adhP* encoding an alcohol dehydrogenase contributes to the reduction of fatty aldehydes to fatty alcohols [36], and its deletion resulted in the accumulation of fatty aldehydes, which caused damage to the cell or the inhibition of enzymes activities by metabolic disorders or potential toxicity [37]. Here, CFS inhibited the expression of *adhP* in *E. coli* ATCC25922 and *S. aureus* ATCC6538, which might be an explanation for the inhibition of bacterial growth caused by CFS from *L. rhamnosus* SCB0119. Secondly, nickel is an essential catalytic cofactor of enzymes allowing organisms to inhabit diverse environmental niches, and its pumping through the cytoplasmic membrane is regulated by NikABCDE [38]. Iron intake positively correlated with the synthesis of some ferritin proteins, such as catalases, whose reduction will cause the intracellular accumulation of H_2_O_2_, induce the oxidative stress response inside the thallus, and bring adverse effects on cellular components such as DNA, proteins, and membrane lipids [26,39,40]. The deletion of *E. coli fepD* caused a reduction in iron intake [41]. Therefore, the downregulation of *nikA*, *nikB*, *nikC*, *nikD*, *nikE*, and *fepD* might result in the damage of nickel and iron homeostasis and then induce the inhibition of bacterial growth in *E. coli* caused by CFS from *L. rhamnosus* SCB0119. Thirdly, the accumulation of amino acids could be attributed to related to protein denaturation and blockage of protein synthesis [42]. The overexpression of *lysC*, *ilvI*, *ilvN*, *leuA*, and *leuB* might increase the production of lysine, leucine, isoleucine, and valine [43], suggesting the presence of CFS from *L. rhamnosus* SCB0119 might promote the biosynthesis of lysine, leucine, isoleucine, and valine and then cause the damage to cell survival in *E. coli*. In addition, the decrease in the expression of *osmY* might be another explanation for the antibacterial activities of CFS from *L. rhamnosus* SCB0119 against *E. coli* because OsmY is a molecular chaperone through a genetic selection that forces cells to optimize unstable protein folding in vivo [44] and positively regulates the integrity of the outer membrane [45]. In *S. aureus* ATCC6538, the inhibition of bacterial growth caused by CFS from *L. rhamnosus* SCB0119 might be due to the changes in protein synthesis by mediating the expression of the ribosome cofactor genes (*frr* and *rbfA*), genes encoding 30S ribosomal proteins (*rpsT*, *rpsD*, *rpsI*, *rpsK*, *rpsM*, *rpsE*, *rpsH*, *rpsJ*, *rpsQ*, *rpsC*, and *rpsS*), genes encoding 50S ribosomal proteins (*rpmG*, *rpmA*, *rplU*, *rpmI*, *rpmJ*, *rplO*, *rplR*, *rplF*, *rplE*, *rplN*, and *rplP*), and genes in the pathway of the aminoacyl-tRNA biosynthesis (*leuS*, *gatB*, *lysS*, *gltX*, *pheT*, and *ileS*), because they were involved in the initiation, elongation, and termination of the assembly of the ribosome [46] and important for protein synthesis [47,48]. Moreover, gene EKM74-RS04795 encodes a single-stranded DNA-binding protein, which is crucial in DNA replication, repair, and recombination [49], was down-regulated by CFS in *S. aureus* ATCC6538, suggesting CFS from *L. rhamnosus* SCB0119 might cause cell damage or death in *S. aureus* by interfering with DNA repair pathways and leading to genomic instability. Besides, the expression of four genes (*atpA*, *atpB*, *atpD*, and *atpE*) encoding ATP synthases hydrolyzing ATP [50] were up-regulated by CFS from *L. rhamnosus* SCB0119 in *S. aureus* ATCC6538, which might result in the decreases of ATP content and alive cells.

Lastly, previous studies demonstrated that the consumption of *L. rhamnosus* strains gave positive results in preventing diarrhea and bacterial vaginosis, restoring normal gastrointestinal microflora, and so on [17]. In addition, when directly mixed with beef, CFS from *Lactobacillus* with stable antimicrobial activity could effectively reduce the microbial load of inoculated pathogens and enhance the quality and safety of beef products [51], and the addition of *L. rhamnosus* into milk could inhibit the growth of *P. fluorescens* and *P. putida* from raw milk and improve the raw milk quality [52]. Therefore, effectiveness in reducing the growth of *E. coli* ATCC25922 and *S. aureus* ATCC6538 suggested that *L. rhamnosus* SCB0119 and its CFS might be directly consumed by humans and animals or mixed with various foods to prevent the infection or contamination of *E. coli* and *S. aureus*, although the findings need further validation of a clinical, practical, and/or industrial scale. In the future, the safety of *L. rhamnosus* SCB0119 and its CFS, active components from CFS, and the evaluation of their application potential in preventing the infection and contamination of *E. coli* and *S. aureus* should be further investigated.

## 4. Material and Methods

### 4.1. Bacterial Strains and Culture Condition

*Lactobacillus rhamnosus* SCB0119 (CGMCC NO: 17618) was isolated from fermented pickles by ourselves and cultivated in De Man Rogosa Sharpe (MRS) medium at 37 °C under anaerobic conditions. *Escherichia coli* ATCC25922 and *Staphylococcus aureus* ATCC6538 were obtained from the American Type Culture Collection (ATCC) and cultivated at 37 °C in LB medium under constant shaking at 180 rpm.

### 4.2. Preparation of Cell-Free Culture Supernatants and Cells of L. rhamnosus SCB0119

Briefly, *L. rhamnosus* SCB0119 was inoculated into MRS medium at a ratio of 3:100 (*v*/*v*) and statically cultured at 37 °C for 60 h. The supernatants were collected, filtered with a 0.22 µm pore filter (Merck-Millipore, Darmstadt, Germany), and served as CFS. Bacterial cells were washed with phosphate buffer saline (PBS) and then resuspended in an equal volume of PBS.

### 4.3. Determination of the Antibacterial Activity of L. rhamnosus SCB0119

The antimicrobial activity of *L. rhamnosus* SCB0119 was evaluated using the agar well diffusion method [53] with some modifications. Briefly, the dilutions of *E. coli* ATCC25922 and *S. aureus* ATCC6538 cells (10^7^ CFU/mL) were added into LB medium with 0.75% agar, respectively. Then, the mixture was poured onto LB agar plates (1.5% agar) with several Oxford cups (6 × 7.8 mm), which were removed when the agar solidified. Aliquots of 100 μL of *L. rhamnosus* SCB0119 culture, CFS, resuspended bacterial cells, and MRS medium were added to the wells and incubated at 37 °C. After 12 h of incubation, the mean diameters of the inhibition zones were recorded as antibacterial activity of *L. rhamnosus* SCB0119 against *E. coli* ATCC25922 and *S. aureus* ATCC6538. The experiments were conducted in triplicate.

### 4.4. Minimum Inhibitory Concentrations of CFS against E. coli and S. aureus 

MICs were determined via the microdilution method in 96-well polystyrene plates (Corning Inc., Corning, NY, USA) [54]. Briefly, CFS was evaporated, dissolved using ultrapure water to 500 mg/mL and stored at −20 °C. Wells containing 100 μL of *E. coli* ATCC25922/*S. aureus* ATCC6538 dilutions (10^7^ CFU/mL) in LB medium were supplemented with different concentrations (0.49–250 mg/mL) of CFS and incubated at 37 °C for 12 h. Optical densities at 600 nm (OD_600_) were read using a Multiskan GO Reader (Thermo Scientific, Waltham, MA, USA). The experiments were conducted in triplicate.

### 4.5. Growth Curve of E. coli and S. aureus after Treatment with CFS

The bacterial growth curve was assayed using the method reported by Yan et al. [55]. Briefly, *E. coli* ATCC25922/*S. aureus* ATCC6538 dilutions (10^7^ CFU/mL) in LB medium were supplemented with 0, 1/2 MIC, and 1 MIC of CFS and incubated in Bioscreen C (Oy Growthcurves Ab Ltd., Turku, Finland), where the temperature was set at 37 °C and the rotating speed was set at 180 rpm. During a 34-h incubation, OD_600_s were read at 2-h intervals. The experiments were conducted in triplicate.

### 4.6. Scanning Electron Microscopy and Transmission Electron Microscopy 

The potential effects of CFS on cell morphology and structure of *E. coli* ATCC25922 and *S. aureus* ATCC6538 were determined by SEM and TEM [56,57]. Briefly, bacterial dilutions (10^7^ CFU/mL) in LB medium were supplemented with 1/2 MIC of CFS and incubated for 10 h at 37 °C by shaking. After centrifugation at 4 °C for 5 min, bacterial cells were collected, washed with PBS (0.1 mM, pH 7.2) three times, fixed overnight with 4% glutaraldehyde, washed with PBS once, and post-fixed in 1% OsO_4_ for 30 min. Some post-fixed cells were dehydrated in a graded ethanol series (50%, 70%, 80%, 90%, and 100%), dried by CO_2_ for 4 h, coated with gold, and observed under a field emission scanning electron microscope (FEI/Talos L120C, Thermo Fisher Scientific, New York, NY, USA). Other post-fixed cells were dyed with uranium acetate overnight, dehydrated in a graded ethanol series (30%, 50%, 70%, 85%, 95%, and 100%), embedded in Spurr resin, polymerized at 55 °C for 48 h, and observed under a transmission electron microscope (ZEISS, Oberkochen, Germany).

### 4.7. Concentrations of Alkaline Phosphatase and Intracellular ATP

Bacterial dilutions of *E. coli* ATCC25922/*S. aureus* ATCC6538 (10^8^ CFU/mL) in PBS for the activity of AKP or 5 mM HEPES buffer for the concentration of intracellular ATP were supplemented with 0, 1/2 MIC, 1 MIC, and 2 MIC of CFS and incubated at 37 °C by shaking. After 1 h, the supernatants or bacteria were collected and used to measure the AKP activity using an AKP kit (Nanjing Jiancheng Bioengineering Institute, Nanjing, China) and ATP content using an ATP detection kit (Beyotime Biotechnology, Shanghai, China) [58] according to the method described by the manufacturer. The concentration of ATP was determined using external calibration curves of the standard ATP solution at different concentrations. Moreover, the protein concentration in the sample was measured using the total protein quantitative determination kit (Nanjing Jiancheng Bioengineering Institute, Nanjing, China), and the concentration of ATP was converted into nmol/mg protein. All of the experiments were conducted in triplicate.

### 4.8. Assay for Outer Membrane Permeability and Inner Membrane Integrity

For the outer membrane permeability, *E. coli* ATCC25922/*S. aureus* ATCC6538 dilutions (10^8^ CFU/mL) in 5 mM HEPES buffer containing 5 mM glucose (pH 7.0) were incubated with 10 µM NPN for 30 min at 25 °C and then supplemented with 0, 1/2 MIC, 1 MIC, and 2 MIC of CFS and incubated at 37 °C by shaking [59]. After 30 min, the fluorescence intensities were recorded using a Varioskan Flash (spectraMax i3x, Molecular Devices, San Francisco, CA, USA) at the excitation/emission wavelengths of 350/420 nm. The experiments were conducted in triplicate.

For the inner membrane integrity, *E. coli* ATCC25922/*S. aureus* ATCC6538 dilutions (10^8^ CFU/mL) in PBS were incubated with 10 µM PI for 30 min at 25 °C and then supplemented with 0, 1/2 MIC, 1 MIC, and 2 MIC of CFS and incubated at 37 °C by shaking [60]. After 1 h, the fluorescence intensities were recorded using the Varioskan Flash at the excitation/emission wavelengths of 535/615 nm. The experiments were conducted in triplicate.

### 4.9. Assays for Membrane Potential, pH Gradient, and Reactive Oxygen Species

ΔΨ, ΔpH, and ROS were assayed with the DiBAC4(3) (Solarbio, Beijing, China) fluorescence probe [61], the BCECF-AM (Yuanye, Shanghai, China) fluorescence probe [62], and a ROS assay kit (Beyotime Biotechnology, Shanghai, China), respectively. Briefly, *E. coli* ATCC25922/*S. aureus* ATCC6538 dilutions (10^8^ CFU/mL) in 5 mM HEPES buffer were incubated with 5 µM DiBAC4(3) dilution, 5 µM BCECF-AM dilution, or 10 µM DCFH-DA dilution incubator for 30 min at 37 °C and then supplemented with 0, 1/2 MIC, 1 MIC, and 2 MIC of CFS and incubated at 37 °C by shaking. After 1 h, the fluorescence intensities were recorded using the Varioskan Flash at the excitation/emission wavelengths of 470/500 nm, 480/535 nm, and 488/525 nm, respectively. All the experiments were conducted in triplicate.

### 4.10. Transcriptome Sequencing and Analysis

Total RNA was extracted from *E. coli* ATCC25922 and *S. aureus* ATCC6538 cells grown in LB medium with 1/2 MIC of CFS for 10 h at 37 °C using TRIzol^®^ Reagent (Invitrogen, Carlsbad, CA, USA) according to the manufacturer’s instructions and genomic DNA was removed using DNase I (TaKaRa, Kyoto, Japan). Then RNA quality was determined using 2100 Bioanalyser (Agilent, Palo Alto, CA, USA) and quantified using the ND-2000 (NanoDrop Technologies, Thermo Fisher Scientific, New York, NY, USA). RNA-seq strand-specific libraries were created using 5 g of total RNA and the TruSeq RNA sample preparation Kit (Illumina, San Diego, CA, USA). rRNA was removed by RiboZero rRNA removal kit (Epicenter, Stockholm, Sweden) and fragmented using fragmentation buffer. The Illumina-indexed adaptors were ligated after cDNA synthesis, end repair, A-base addition, and ligation according to Illumina’s protocol. cDNA target fragments of 200–300 bp were selected on 2% Low Range Ultra Agarose followed by PCR amplified using Phusion DNA polymerase (NEB) for 15 PCR cycles. Paired-end libraries were sequenced by Illumina NovaSeq 6000 sequencing (150 bp × 2, Shanghai BIOZERON Co., Ltd, Shanghai, China) after being quantified by TBS380. The sequencing data were deposited in NCBI Sequence Read Archive (http://www.ncbi.nlm.nih.gov/Traces/sra) under the accession numbers SRP395161 for *E. coli* ATCC25922 and SRP394988 for *S. aureus* ATCC6538.

Clean data were mapped to the data from the *E. coli* genome (NCBI accession: ASM301845v1) and the *S. aureus* genome (NCBI accession: ASM394542v1), respectively. The expression level of each gene was evaluated by calculating the fragments per kilobase per million fragments mapped (FPKM), and edgeR (https://bioconductor.org/packages/release/bioc/html/edgeR.html) was used for differential expression analysis. To compare the differential gene expression among the libraries, the false discovery rate (FDR) ≤0.05 and the absolute value of the log_2_ ratio ≥1 were used as the threshold to screen the DEGs. Functional classification and pathway analysis of DEGs were performed using GO functional enrichment (Goatools, https://github.com/tanghaibao/Goatools) and KEGG pathway analysis (KOBAS, http://kobas.cbi.pku.edu.cn/kobas3), respectively. The experiments were conducted in twice.

### 4.11. qRT-PCR

Total RNA was extracted from *E. coli* ATCC25922 and *S. aureus* ATCC6538 cells grown in LB medium with 1/2 MIC of CFS for 10 h at 37 °C using TRIzol^®^ Reagent (Invitrogen, Carlsbad, CA, USA), detected by agarose gel electrophoresis, quantified using the ND-2000 (NanoDrop Technologies, Thermo Fisher Scientific, New York, NY, USA), and transcribed reversely into cDNA with PrimeScript RT reagent kit (TaKaRa, Kyoto, Japan). Tenfold dilutions of each cDNA were used as templates to analyze the expression of tested genes via qRT-PCR with paired primers (Table S1) using SYBR^®^ Premix Ex Taq™ (TaKaRa, Kyoto, Japan). The reaction cycling consisted of initial denaturation at 95 °C for 30 s, followed by 40 cycles of denaturation at 95 °C for 30 s, annealing and extension at 60 °C for 34 s, and ended up with a dissolution curve. The analysis of the dissolution curve evaluated the specificity of the primers. The 16 s rRNA was used as an internal standard. The relative transcription level of each gene was defined as the ratio of its transcript in cells treated with CFS over that without CFS using the 2^−ΔΔCt^ method, where Ct means the threshold cycle, ΔCt is equal to the difference in threshold cycles for target and reference (Ct_x_ − Ct_r_), and ΔΔCt equals (ΔCt_in CFS group_ − ΔCt_in control group_) [63]. The experiments were conducted in triplicate.

### 4.12. Data Analysis

Results from replicates were expressed as mean ± standard deviation (SD), and the data analysis was subjected to a one-factor analysis of variance, followed by Tukey’s HSD test. *p* < 0.05 was considered a significant difference in all experiments.

## 5. Conclusions

*Lactobacillus rhamnosus* SCB0119 could inhibit the growth and induce cell death of *E. coli* and *S. aureus*, and its antibacterial activity was mainly attributed to the CFS. Its CFS could induce oxidative stress involving ROS and cause damage to the integrity and permeability of the cell wall and membrane, which resulted in the release of the cell contents. Moreover, the transcriptomic analysis showed that CFS aimed at the expression of genes involved in fatty acid degradation, ion transport, and the biosynthesis of amino acids in *E. coli* and affected the expression of genes involved in fatty acid degradation, protein synthesis, DNA replication, and ATP hydrolysis in *S. aureus*. These findings demonstrated *L. rhamnosus* SCB0119 and its CFS have the potential for use as a biocontrol agent against pathogens, including *E. coli* and *S. aureus*. 

## Figures and Tables

**Table 1:** Gene annotation and KEGG pathway of the differentially expressed genes in *E. coli* ATCC25922 in response to CFS from *L. rhamnosus* SCB0119.

Gene ID	RPKM Control	RPKM CFS	log2 Ratio	Gene Annotation
Fatty acid degradation				
C7A06_RS17240	9.40 ± 0.16	2.62 ± 0.05	−1.83961	*adhP*; alcohol dehydrogenase AdhP
Biosynthesis of amino acids				
C7A06_RS27640	4.88 ± 1.53	21.23 ± 2.32	2.119947	*ilvN*; acetolactate synthase small subunit
C7A06_RS27645	5.18 ± 0.68	24.79 ± 0.65	2.259582	*ilvI*; acetolactate synthase 3 large subunit
C7A06_RS27660	17.62 ± 0.97	58.19 ± 0.51	1.723499	*leuA*; 2-isopropylmalate synthase
C7A06_RS27665	15.63 ± 2.17	48.10 ± 7.45	1.621654	*leuB*; 3-isopropylmalate dehydrogenase
C7A06_RS30700	15.96 ± 0.02	155.56 ± 1.24	3.284926	*lysC*; lysine-sensitive aspartokinase 3
ABC transporters				
C7A06_RS01665	14.27 ± 0.12	6.44 ± 0.43	−1.14856	*nikE*; nickel import ATP-binding protein NikE
C7A06_RS01670	39.57 ± 0.39	15.70 ± 1.38	−1.33379	*nikD*; nickel import ATP-binding protein NikD
C7A06_RS01675	43.38 ± 0.05	11.55 ± 1.74	−1.90906	*nikC*; nickel ABC transporter permease subunit NikC
C7A06_RS01680	39.74 ± 2.21	10.25 ± 2.12	−1.95437	*nikB*; nickel ABC transporter permease subunit NikB
C7A06_RS01685	10.05 ± 1.12	1.86 ± 0.26	−2.43055	*nikA*; nickel ABC transporter substrate-binding protein
C7A06_RS01780	0.15 ± 0.08	1.01 ± 0.42	2.788886	*livH*; ABC transporter permease LivH
C7A06_RS01790	4.29 ± 0.27	14.90 ± 1.90	1.794947	*livG*;ABC transporter ATP-binding protein LivG
C7A06_RS24375	3.74 ± 0.98	1.53 ± 0.65	−1.29111	*fepD*; Fe^3+^ -siderophore ABC transporter permease

**Table 2:** Gene annotation and KEGG pathway of the differentially expressed genes in *S. aureus* ATCC6538 in response to CFS from *L. rhamnosus* SCB0119.

Gene ID	RPKM Control	RPKM CFS	log2 Ratio	Gene Annotation
Fatty acid degradation				
EKM74_RS11915	1832.07 ± 490.15	697.14 ± 121.09	−1.39396	*adhP*; alcohol dehydrogenase
DNA replication				
EKM74_RS04795	85.2 ± 44.94	39.85 ± 1.91	−1.09611	single-stranded DNA-binding protein
Ribosome				
EKM74_RS01000	77.06 ± 19.54	474.46 ± 30.77	2.622215	*accC*; acetyl-CoA carboxylase biotin carboxylase subunit
EKM74_RS01125	36.7 ± 12.69	642.43 ± 184.29	4.129847	*rpmG*; 50S ribosomal protein L33
EKM74_RS01310	21.83 ± 0.96	168.95 ± 53.18	2.951911	*rpsT*; 30S ribosomal protein S20
EKM74_RS01660	458.35 ± 97.45	2756.92 ± 263.1	2.588527	*rpmA*; 50S ribosomal protein L27
EKM74_RS01670	102.05 ± 29.64	4057 ± 488.97	5.313122	*rplU*; 50S ribosomal protein L21
EKM74_RS01855	19.42 ± 10.05	621.08 ± 94.23	4.999194	*rplT*; 50S ribosomal protein L20
EKM74_RS01860	51.94 ± 21.24	5478.49 ± 45.03	6.720704	*rpmI*; 50S ribosomal protein L35
EKM74_RS02080	80.66 ± 17.27	3451.94 ± 642.32	5.419314	*rpsD*; 30S ribosomal protein S4
EKM74_RS05465	190.13 ± 11.18	3317.01 ± 500.37	4.124828	*rpsI*; 30S ribosomal protein S9
EKM74_RS05470	150.17 ± 31.96	5880.76 ± 17.12	5.291357	*rplM*; 50S ribosomal protein L13
EKM74_RS05495	122.74 ± 4.32	3341.82 ± 309.69	4.766903	*rplQ*; 50S ribosomal protein L17
EKM74_RS05505	287.56 ± 78.6	2321.16 ± 704.2	3.01292	*rpsK*; 30S ribosomal protein S11
EKM74_RS05510	582.25 ± 5.82	10,572.69 ± 1079.55	4.182553	*rpsM*; 30S ribosomal protein S13
EKM74_RS05515	259.55 ± 26.92	8724.69 ± 798.78	5.071017	*rpmJ*; 50S ribosomal protein L36
EKM74_RS05535	328.44 ± 51.85	2770.35 ± 239.31	3.076378	*rplO*; 50S ribosomal protein L15
EKM74_RS05540	370.08 ± 105.31	13,564.79 ± 1089.74	5.195877	*rpmD*; 50S ribosomal protein L30
EKM74_RS05545	183.9 ± 22.05	4254.61 ± 754.45	4.532035	*rpsE*; 30S ribosomal protein S5
EKM74_RS05550	205.01 ± 26.43	9035.82 ± 328.78	5.461917	*rplR*; 50S ribosomal protein L18
EKM74_RS05555	81.16 ± 4.22	1809.37 ± 428.25	4.478643	*rplF*; 50S ribosomal protein L6
EKM74_RS05560	98.44 ± 3.27	3014.88 ± 247.03	4.93666	*rpsH*; 30S ribosomal protein S8
EKM74_RS05570	126.29 ± 2.95	4279.22 ± 391.93	5.082565	*rplE*; 50S ribosomal protein L5
EKM74_RS05575	138.6 ± 11.64	4600.35 ± 70.63	5.052719	*rplX*; 50S ribosomal protein L24
EKM74_RS05580	181.27 ± 11.93	4136.6 ± 837.23	4.512211	*rplN*; 50S ribosomal protein L14
EKM74_RS05585	140.46 ± 16.11	4981.9 ± 113.47	5.148408	*rpsQ*; 30S ribosomal protein S17
EKM74_RS05590	154.79 ± 34.38	4723.29 ± 418.72	4.931395	*rpmC*; 50S ribosomal protein L29
EKM74_RS05595	194.99 ± 8.39	3442.61 ± 618.09	4.142009	*rplP*; 50S ribosomal protein L16
EKM74_RS05600	333.68 ± 100.38	6017.46 ± 493.68	4.172617	*rpsC*; 30S ribosomal protein S3
EKM74_RS05605	267.84 ± 59.12	9325.05 ± 0.95	5.121682	*rplV*; 50S ribosomal protein L22
EKM74_RS05610	210.47 ± 4.59	7349.55 ± 191.55	5.125963	*rpsS*; 30S ribosomal protein S19
EKM74_RS05615	231.04 ± 32.27	4313.16 ± 576.59	4.222508	*rplB*; 50S ribosomal protein L2
EKM74_RS05620	87.68 ± 7.24	1918.56 ± 543.34	4.451662	*rplW*; 50S ribosomal protein L23
EKM74_RS05625	145.16 ± 12.67	3427.73 ± 563.02	4.561522	*rplD*; 50S ribosomal protein L4
EKM74_RS05630	188.98 ± 15.87	7819.97 ± 221.75	5.37082	*rplC*; 50S ribosomal protein L3
EKM74_RS05635	294.61 ± 86.56	9259.91 ± 978.26	4.97412	*rpsJ*; 30S ribosomal protein S10
EKM74_RS07510	151.39 ± 9.3	705.28 ± 120.89	2.219912	*rplI*; 50S ribosomal protein L9
EKM74_RS07590	114.58 ± 17.68	1144.54 ± 138.16	3.320336	*rpmH*; 50S ribosomal protein L34
EKM74_RS10450	236.72 ± 37.3	7542.84 ± 237.11	4.993827	*rpsF*; 30S ribosomal protein S6
EKM74_RS10460	55.84 ± 4.69	531.48 ± 99.45	3.250636	*rpsR*; 30S ribosomal protein S18
EKM74_RS11530	51.86 ± 16.74	493.86 ± 62.28	3.251456	*rpmG*; 50S ribosomal protein L33
EKM74_RS11545	230.05 ± 33.59	6745.81 ± 496	4.873994	*rplK*; 50S ribosomal protein L11
EKM74_RS11550	89.54 ± 9.77	1345.42 ± 304.26	3.909295	*rplA*; 50S ribosomal protein L1
EKM74_RS11560	75.58 ± 8	6432.15 ± 34.02	6.411134	*rplJ*; 50S ribosomal protein L10
EKM74_RS11565	38.15 ± 13.06	2351.81 ± 364.44	5.945945	*rplL*; 50S ribosomal protein L7/L12
EKM74_RS11590	185.09 ± 16.78	6759.64 ± 1120.81	5.190643	*rpsL*; 30S ribosomal protein S12
EKM74_RS11595	156.22 ± 21.95	3889.22 ± 770.69	4.637864	*rpsG*; 30S ribosomal protein S7
EKM74_RS14380	74.47 ± 6.98	956.98 ± 89.75	3.683755	*rpmF*; 50S ribosomal protein L32
EKM74_RS15340	44.89 ± 6.07	387.7 ± 59.81	3.110434	*rpsP*; 30S ribosomal protein S16
EKM74_RS15355	56.77 ± 3.55	839.03 ± 212.16	3.885462	*rplS*; 50S ribosomal protein L19
EKM74_RS15435	112.72 ± 30.02	3718.42 ± 314.52	5.043867	*rpsB*; 30S ribosomal protein S2
EKM74_RS15530	178.88 ± 2.05	2219.27 ± 90.62	3.633007	*rpsO*; 30S ribosomal protein S15
Aminoacyl-tRNA biosynthesis				
EKM74_RS02305	35.37 ± 21.65	401.03 ± 43.46	3.503254	*leuS*; leucine-tRNA ligase
EKM74_RS03605	63.4 ± 16.82	997.64 ± 109.83	3.975903	*gatB*; Asp-tRNA (Asn)/Glu-tRNA (Gln) amidotransferase subunit
EKM74_RS11370	90.84 ± 8.58	509.92 ± 53.53	2.488778	*lysS*; lysine-tRNA ligase
EKM74_RS11490	107.77 ± 37.9	605.02 ± 32.1	2.489052	*gltX*; glutamate-tRNA ligase
EKM74_RS14775	34.9 ± 14.25	239.53 ± 20.28	2.77887	*pheT*; phenylalanine-tRNA ligase subunit beta
EKM74_RS15105	30.17 ± 17.78	273.57 ± 29.78	3.180715	*ileS*; isoleucine-tRNA ligase
Oxidative phosphorylation				
EKM74_RS04830	170.47 ± 3.15	3033.08 ± 240.81	4.153207183	*atpD*; F0-F1 ATP synthase subunit beta
EKM74_RS04840	97.18 ± 1.86	1251.21 ± 170.89	3.686521282	*atpA*; F0-F1 ATP synthase subunit alpha
EKM74_RS04855	241.14 ± 56.65	1679.25 ± 147.57	2.799862175	*atpE*; F0-F1 ATP synthase subunit C
EKM74_RS04860	239.15 ± 52.69	1550.63 ± 98.56	2.696892312	*atpB*; F0-F1 ATP synthase subunit A

## Data Availability

All data supporting the findings of this study are available from the corresponding author upon reasonable request.

## Supplementary Materials

The following supporting information can be downloaded at: https://www.mdpi.com/article/10.3390/ijms232315159/s1.

## Abbreviations

CFS	cell-free culture supernatant
LB	Luria Bertani
MICs	minimum inhibitory concentrations
SEM	scanning electron microscopy
TEM	transmission electron microscopy
AKP	alkaline phosphatase
NPN	1-N-phenylnaphthylamine
PI	propidium iodide
ΔΨ	membrane potential
ΔpH	pH gradient
ROS	reactive oxygen species
DEGs	differentially expressed genes
GO	gene ontology
KEGG	Kyoto encyclopedia of genes and genomes
MRS	De Man Rogosa Sharpe
PBS	phosphate buffer saline
FPKM	fragments per kilobase per million fragments mapped
ATCC	American Type Culture Collection
SD	standard deviation
